# Neurological Mystery Post Travel: An Atypical Presentation and Partial Recovery With Immunotherapy

**DOI:** 10.7759/cureus.79611

**Published:** 2025-02-25

**Authors:** Prasobh Pootharamanna Variyath Mukundan, Kavya Rajendran, John Dixon

**Affiliations:** 1 Critical Care, Epsom and St Helier University Hospitals, London, GBR; 2 General Medicine, Epsom and St Helier University Hospitals, London, GBR

**Keywords:** atypical neurological syndrome, autoimmune neuropathy, guillain-barré syndrome mimic, neurological diagnostic uncertainty, neurology and critical care

## Abstract

This case highlights the diagnostic challenges in atypical neurological presentations and the role of empirical immunotherapy despite the absence of a definitive diagnosis. We present a South Indian woman in her 40s who developed progressive lower limb weakness and respiratory compromise after recent international travel. Initially suspected to have Guillain-Barré syndrome (GBS), extensive investigations, including electromyography (EMG), nerve conduction studies (NCS), and cerebrospinal fluid (CSF) analysis, failed to confirm a clear diagnosis.

Despite this uncertainty, empirical intravenous immunoglobulin (IVIG) therapy resulted in clinical improvement, supporting the likelihood of an immune-mediated process. Notably, the patient had been experiencing an ongoing flare-up of vitiligo, an autoimmune disease characterized by immune-mediated destruction of melanocytes, for approximately six months leading up to and during the onset of neurological symptoms, suggesting heightened autoimmune activity around the period of symptom onset. Additionally, the patient's South Indian ethnicity is relevant, as autoimmune disorders, including neurological conditions, may have distinct presentations and prevalence among South Asian populations due to genetic and environmental factors.

The patient’s prolonged ICU stay, need for mechanical ventilation, and complications such as stridor and vocal cord dysfunction underscore the complexity of managing undifferentiated neurological cases.

This case serves as an important educational tool, demonstrating the limitations of standard diagnostic criteria in rare or evolving neurological syndromes, the importance of empirical immunotherapy in suspected immune-mediated conditions, and the need for a multidisciplinary approach with careful follow-up. By highlighting the intersection between neurology, immunology, and critical care, this case reinforces the importance of clinical judgment and individualized treatment strategies when conventional diagnostics fall short.

## Introduction

Diagnosing acute neurological deficits with respiratory compromise is often challenging, particularly when classical diagnostic criteria are not met. Guillain-Barré syndrome (GBS) is a common post-infectious neuropathy characterized by ascending weakness and respiratory muscle involvement, typically confirmed by electrophysiological evidence of peripheral nerve demyelination or elevated protein levels in cerebrospinal fluid (CSF). However, not all patients fit this typical presentation or diagnostic pattern, creating uncertainty in clinical management.

This diagnostic complexity is particularly prominent in patients presenting after recent international travel, where infectious, post-infectious, autoimmune, and even environmental factors must all be considered. Additionally, the coexistence of autoimmune conditions, such as vitiligo, may indicate heightened systemic autoimmune activity, further complicating the diagnostic picture. In South Asian populations, genetic and environmental predispositions contribute to distinct presentations of autoimmune and neurological conditions, underscoring the necessity for individualized diagnostic and therapeutic strategies.

We present a case of a South Indian woman in her 40s who developed rapidly progressive neurological deficits beginning with lower limb weakness that ascended to involve the upper limbs, accompanied by facial muscle weakness, swallowing difficulties, and blurred vision. The rapid progression culminated in severe respiratory compromise requiring mechanical ventilation and tracheostomy. Despite extensive investigations, including nerve conduction studies (NCS), electromyography (EMG), CSF analysis, and autoimmune screening, failing to confirm a definitive diagnosis, the patient's clinical improvement following empirical intravenous immunoglobulin (IVIG) therapy strongly suggested an underlying immune-mediated process.

The primary aim of this case report is to highlight the complexities encountered in diagnosing atypical neurological syndromes in post-travel patients who exhibit clinical features suggestive of an immune-mediated neuropathy, yet fail to fulfil classical diagnostic criteria. Thus, this case serves as an important educational tool, illustrating the limitations of standard diagnostic algorithms, emphasizing the critical role of empirical immunotherapy when confronted with diagnostic uncertainty, and demonstrating the importance of a multidisciplinary approach involving neurology, immunology, and critical care specialists in managing diagnostically challenging neurological conditions.

## Case presentation

Patient background

A South Indian woman in her 40s was admitted to the hospital presenting with progressive neurological symptoms following recent international travel to the Maldives. Her initial symptoms included diarrhoea approximately two weeks prior to admission, followed by progressive bilateral lower limb numbness and weakness beginning about 10 days before hospitalisation. These symptoms gradually ascended to involve upper limbs and cranial nerves, causing facial weakness, swallowing difficulty, and blurry vision just prior to her ICU admission.

Her medical history was significant for prediabetes, polycystic ovarian syndrome (PCOS), dyslipidaemia, arthritis, spontaneous chronic urticaria, and vitiligo, the latter associated with an exacerbation approximately six months before the neurological onset. She had previously undergone a salpingectomy approximately 20 years prior for an ectopic pregnancy. Additionally, she had a benign congenital mole.

Initial admission

On initial evaluation, the patient exhibited progressive bilateral lower limb weakness, with preserved deep tendon reflexes and no overt sensory deficits. Neurological symptoms rapidly evolved, progressing within a few days to involve bilateral upper limb weakness, facial muscle weakness, dysphagia, blurred vision, and paraesthesia around the mouth. Spirometry revealed a significantly reduced forced expiratory volume in one second (FEV1) of 1.15 L initially, which further decreased to 0.53 L over the next four days, reflecting rapidly progressive respiratory compromise.

Early neurological consultation was obtained, and diagnostic evaluations, including lumbar puncture (LP), magnetic resonance imaging (MRI) of the brain and cervical spine, electromyography (EMG), and nerve conduction studies (NCS), were performed within the first week. MRI and LP returned unremarkable findings, and initial EMG/NCS studies did not support classical GBS. Nonetheless, given the clinical presentation, recent diarrhoeal illness, rapidly progressive flaccid weakness with ascending pattern, cranial nerve involvement, and respiratory failure, an immune-mediated neuropathy, specifically an atypical variant of GBS or acute motor axonal neuropathy (AMAN), was strongly suspected. Neuromuscular junction (NMJ) disorders such as myasthenia gravis (MG) and Lambert-Eaton myasthenic syndrome (LEMS) were also initially considered due to cranial nerve involvement and rapid symptom progression.

Based on the high clinical suspicion of an immune-mediated process, empirical IVIG therapy was initiated five days after admission (15 days from symptom onset), at a dose of 2 g/kg (total 90 g) administered over five days. Despite initiation of IVIG, respiratory function continued to deteriorate, necessitating intubation, mechanical ventilation, and transfer to the ICU seven days after admission.

ICU course

Upon transfer, the patient underwent further diagnostic evaluation and treatment.

Days One to Seven

Initial EMG and NCS did not support the classical diagnosis of GBS. Despite diagnostic uncertainty, IVIG therapy was continued based on clinical suspicion. Anti-acetylcholine receptor (AChR) and anti-muscle-specific kinase (anti-MuSK) antibodies returned negative, significantly reducing suspicion for NMJ disorders such as MG or LEMS.

**Figure 1:**
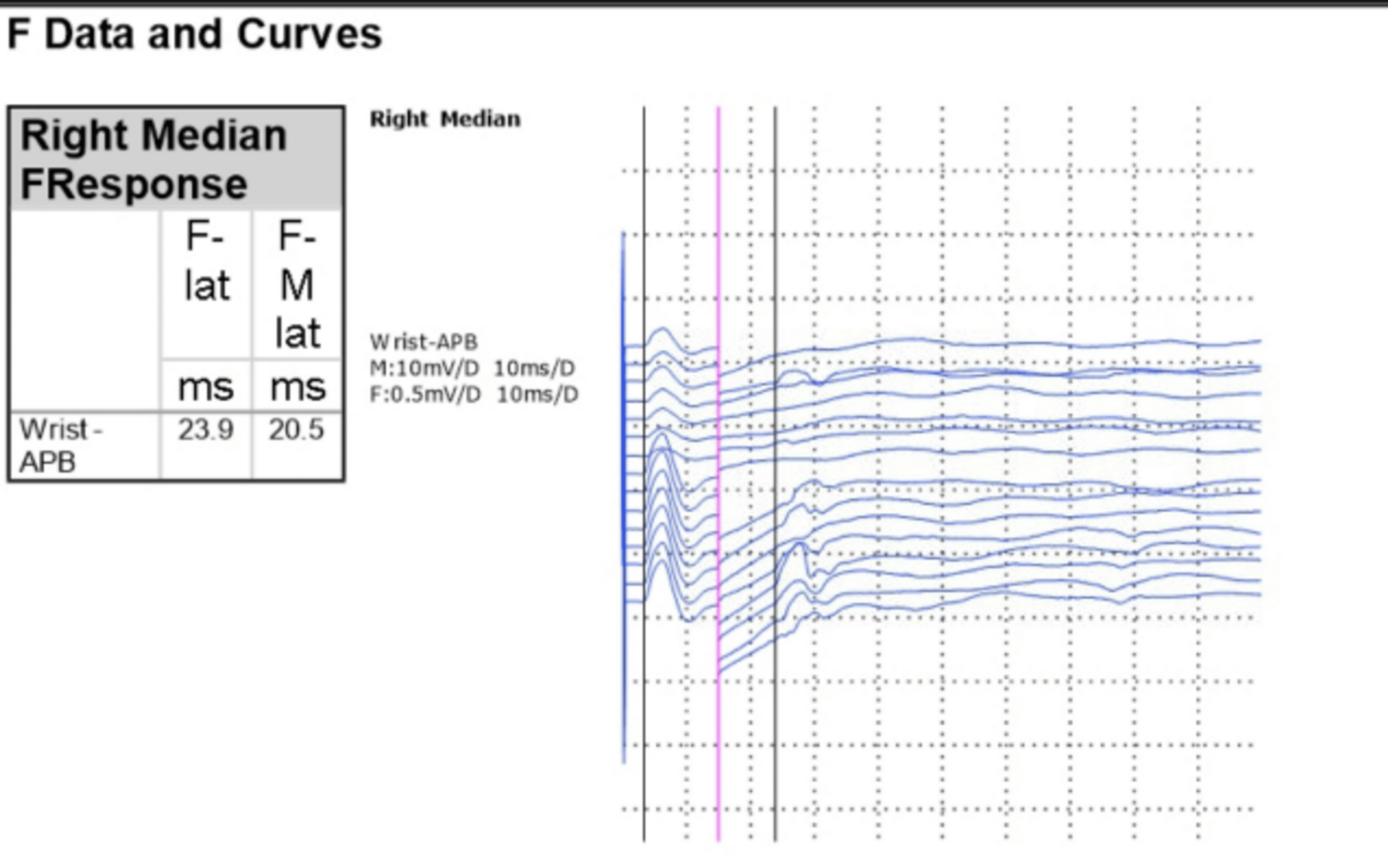
Right median nerve F-wave responses post-intravenous immunoglobulin treatment demonstrating normalized persistence and latency. APB: abductor pollicis brevis.

A repeat EMG/NCS evaluation, including repetitive nerve stimulation of three different nerves and single-fibre EMG of the orbicularis oculi muscle, was conducted after the completion of IVIG therapy (Figure 1 and Table 1). This follow-up assessment confirmed the continued absence of peripheral nerve demyelination and NMJ dysfunction, conclusively excluding GBS and MG. Importantly, the subtle initial abnormality, i.e., reduced persistence of late F-wave responses in the right median nerve, had completely resolved post-IVIG therapy (Figure 1), suggesting a reduction in inflammatory activity and supporting an IVIG-responsive immune-mediated neuropathy.

**Table 1:** Single-fibre EMG of orbicularis oculi muscle. EMG: electromyography; MCD: mean consecutive difference; MIDI: mean inter-discharge interval.

No.	Jitter (us)	MCD (us)	MIDI (ms)	Block	Block %
1	38.0	38.0	33.0	☐	50.7
2	26.4	26.4	31.7	☐	83.5
3	28.5	28.5	130	☐	10.0
4	12.6	12.6	47.5	☐	0
5	17.2	17.2	225	☐	11.5
6	21.8	21.8	225	☐	0
7	15.4	15.4	225	☐	3.8
8	18.7	18.7	70.3	☐	2.4
9	14.6	14.6	46.3	☐	40.0
10	20.0	20.0	48.6	☐	54.3
11	12.8	12.8	48.6	☐	42.9
12	34.0	34.0	48.6	☐	28.6
13	13.3	13.3	69.7	☐	3.3
14	11.6	11.6	44.2	☐	25.4
15	30.1	30.1	44.2	☐	0
16	25.3	25.3	31.1	☐	59.1
17	9.5	9.5	117	☐	14.0
18	13.1	13.1	23.9	☐	57.1
19	13.9	13.9	23.9	☐	58.2
Mean	19.8	19.8	-	-	-

Despite these electrophysiological improvements, the patient continued to experience significant fatigue, delaying extubation. Spontaneous breathing trials (SBT) were attempted twice but were unsuccessful due to persistent respiratory muscle weakness.

Days Seven to Fourteen

**Figure 2:**
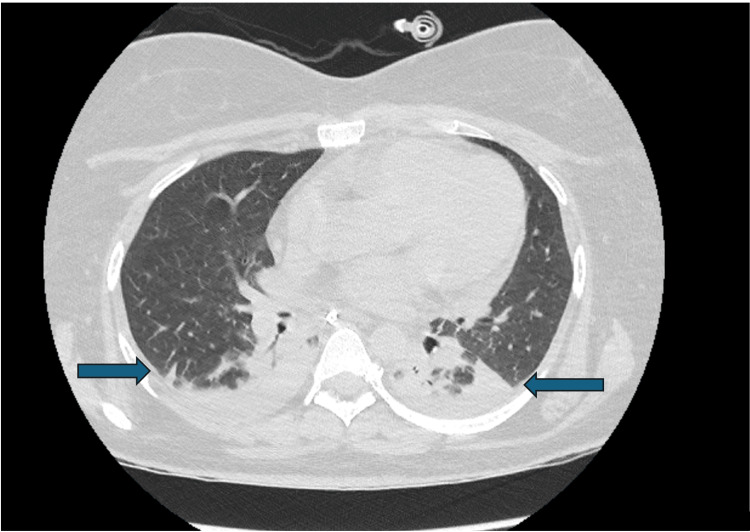
CT scan of the chest. CT scan of the chest demonstrating aspiration pneumonitis, bilateral lower lobe collapse, and small pleural effusions (indicated by arrows). The CT of the neck was performed but showed no significant findings.

The patient was extubated but developed significant stridor, necessitating prompt re-intubation. A CT scan of the neck and chest (Figure 2) subsequently revealed aspiration pneumonitis, bilateral lower lobe collapse, and small pleural effusions, likely resulting from prolonged mechanical ventilation and ICU positioning. MRI of the brain and cervical spine was repeated and continued to show no evidence of encephalitis or demyelination; however, pansinusitis and mastoiditis were noted, consistent with prolonged ICU stay and immobilization. A tracheostomy was subsequently performed to facilitate ongoing ventilatory support and airway management.

CSF results were obtained on day four and day 19 (Table 2).

**Table 2:** CSF findings (multiple lumbar punctures). CSF: cerebrospinal fluid; PCR: polymerase chain reaction.

Day	CSF analysis	Findings
Day 4	Initial lumbar puncture	Clear fluid, normal cell count, normal protein (0.20 mg/dl), normal glucose.
	CSF viral PCR tests	Negative for herpes simplex virus (HSV), varicella-zoster virus (VZV), and Enterovirus.
Day 19	Follow-up CSF analysis	Clear fluid, normal cell count, normal protein (0.20 mg/dl), normal glucose; negative extended screening including Zika virus and Rickettsia.
	Diagnostic interpretation	Excluded infectious encephalitis, further complicating diagnostic evaluation.

Days 14 to 21

Given that GBS and NMJ disorders had been conclusively excluded, an autoimmune aetiology remained strongly suspected. Therefore, a second course of IVIG was administered (total dose of 2 g/kg, 90 g IV over five days), approximately four weeks after the onset of initial symptoms. Following this second IVIG treatment, the patient showed gradual clinical improvement characterized by enhanced motor strength, improved mobilization, and respiratory stability. She was subsequently successfully weaned from mechanical ventilation and her tracheostomy was decannulated.

Extensive autoimmune and infectious screening was conducted to rule out potential aetiologies. The key investigations and their interpretations are summarized in Table 3.

**Table 3:** Investigations. HSV: herpes simplex virus; VZV: varicella-zoster virus; CNS: central nervous system; AchR: acetylcholine receptor; MuSK: muscle-specific kinase; LGI1: leucine-rich glioma-inactivated 1; NMDA: N-methyl-D-aspartate; VGCC: voltage-gated calcium channels; GM1: ganglioside; ANA: antinuclear antibody; RF: rheumatoid factor; anti-CCP: anti-cyclic citrullinated peptide; P-ANCA: perinuclear antineutrophil cytoplasmic antibody.

Diagnostic test	Result	Interpretation
Autoimmune encephalitis & neuromuscular panel	AchR, MuSK, LGI1, contactin-2, NMDA, VGCC, GM1 antibodies: negative	Excluded neuromuscular junction and common autoimmune encephalitis disorders
Autoimmune markers	ANA, RF, anti-CCP, proteinase-3, myeloperoxidase antibodies: normal; P-ANCA: positive	Non-specific finding; no evidence of vasculitis
Cerebrospinal fluid (CSF) and blood polymerase chain reaction (PCR)	Negative for HSV, VZV, Enterovirus, Zika	Excluded common viral infections
Serum electrophoresis	No paraproteins detected	Excluded monoclonal gammopathy/malignancy
Imaging (MRI & CT)	MRI brain/spine: normal; CT chest: aspiration pneumonitis, pleural effusions, lobe collapse	No structural CNS lesions; complications from prolonged intubation
Sputum culture	*Staphylococcus aureus* cultured	Likely a contaminant; clinically insignificant

Given the complexity of this case, multiple differential diagnoses were carefully evaluated, including GBS, chronic inflammatory demyelinating polyneuropathy (CIDP), AMAN, autoimmune encephalitis, NMJ disorders (MG and LEMS), post-infectious autoimmune neuropathy, and functional neurological disorder (FND). Table 4 summarizes these differentials, the rationale behind their initial consideration, and the critical factors leading to their eventual exclusion or inclusion.

**Table 4:** Differential diagnosis. CNS: central nervous system; EMG: electromyography; NCS: nerve conduction study; CSF: cerebrospinal fluid; IVIG: IV immunoglobulin; MG: myasthenia gravis; LEMS: Lambert-Eaton myasthenic syndrome; AchR: acetylcholine receptor; MuSK: muscle-specific kinase.

Diagnosis	Reason for consideration	Supporting/excluding factors
Guillain-Barré syndrome (GBS)	Initially suspected due to acute ascending weakness, cranial nerve involvement, and respiratory compromise post infection.	Excluded due to the absence of demyelinating features on repeated EMG/NCS studies and consistently normal CSF findings.
Chronic inflammatory demyelinating polyneuropathy (CIDP)	Considered due to progressive weakness pattern.	Excluded due to acute onset, lack of chronic progression, and absence of characteristic EMG/NCS findings.
Acute motor axonal neuropathy (AMAN)	A GBS variant presenting predominantly with motor weakness and minimal sensory involvement.	Considered likely due to clinical presentation and response to IVIG, though definitive electrophysiological findings were lacking.
Autoimmune encephalitis	Given the concurrent flare-up of vitiligo, a potential autoimmune process affecting CNS was considered.	Excluded due to absence of seizures, normal MRI imaging, normal CSF, and negative autoimmune encephalitis panel results.
Neuromuscular junction disorders (MG, LEMS)	Considered due to prominent cranial nerve involvement, fluctuating weakness, and rapid respiratory deterioration.	Excluded by negative AchR and MuSK antibodies, normal repetitive nerve stimulation and single-fibre EMG, and lack of characteristic clinical features of MG/LEMS.
Post-infectious autoimmune neuropathy	Likely diagnosis due to recent diarrhoeal illness, travel history, progressive ascending weakness, cranial nerve involvement, and IVIG responsiveness.	Supported by clinical response to IVIG despite negative specific antibody markers; remained a strong differential due to compatible clinical profile.
Functional neurological disorder (FND)	Considered due to non-specific neurological features potentially mimicking organic disease.	Excluded due to severity of respiratory compromise, clear objective weakness, and improvement post IVIG—findings atypical of FND.

Treatment

The patient received a second course of IVIG at a dose of 2 g/kg (total 90 g) divided over five days. IVIG was selected as the primary treatment due to its established efficacy across a broad range of immune-mediated neuropathies, favourable safety profile, and ready accessibility in our clinical setting. Corticosteroids were not administered, as their role remains uncertain in acute immune-mediated neuropathies, with limited supporting evidence for efficacy.

Plasmapheresis was considered given its comparable efficacy in treating severe immune-mediated neuropathies; however, IVIG was favoured due to logistical constraints and the patient's initial partial but encouraging clinical response to IVIG.

Following the second IVIG course, the patient demonstrated significant improvements in muscle strength and respiratory function. She was successfully weaned from ventilatory support and decannulated approximately four weeks after initial hospitalization. Multidisciplinary supportive care, including physical therapy, nutritional support, regular speech and language therapy assessments, and tracheostomy care, played a critical role in her recovery. Following decannulation, she successfully transitioned to oral intake.

Outcomes and follow-up

The patient was discharged from the hospital after approximately five weeks, including nearly four weeks of ICU care, with scheduled outpatient follow-up by neurology and speech therapy. At the most recent neurology review, the patient demonstrated significant neurological recovery, with a near-complete resolution of motor weakness, facial muscle involvement, and visual disturbances. She is now mobilizing independently and has returned to near-baseline functional status.

However, during subsequent speech therapy evaluation, she was noted to have developed left vocal cord immobility. This complication is likely related to prolonged intubation and the challenging second intubation, which occurred due to laryngeal oedema during her initial extubation attempt. The immobile vocal cord resulted in symptoms of a weak, hoarse voice and mild exertional stridor, secondary to reduced airway patency. Treatment options were discussed with the patient, including potential surgical repositioning of the vocal cord, although it was noted this procedure might further impact voice quality. She has been referred to an airway specialist for additional evaluation and definitive management.

Given the isolated nature of this episode, long-term immunotherapy was not initiated. However, the patient remains under close neurology follow-up, with clear plans for monitoring, reassessment, and further investigations should symptoms recur. The case highlights the diagnostic uncertainty often present in atypical immune-mediated neurological syndromes, where the clinical response to empirical immunotherapy can guide ongoing management despite inconclusive biomarkers.

## Discussion

This case illustrates the diagnostic challenges encountered when patients present with rapidly progressive neurological deficits in the absence of typical electrophysiological findings, such as those seen in GBS. GBS is classically characterized as a post-infectious autoimmune polyneuropathy often triggered by preceding gastrointestinal or respiratory illnesses [1,2]. Although this patient exhibited clinical features compatible with GBS, including ascending paralysis, cranial nerve involvement, and respiratory compromise, the absence of typical electrophysiological findings, specifically peripheral nerve demyelination on NCS and EMG, questioned this diagnosis and raised the possibility of alternative diagnoses, including NMJ disorders or primary muscle pathology [2]. However, negative results for AChR and MuSK antibodies, normal serum creatine kinase (CK) levels, and normal repetitive nerve stimulation studies and single-fibre EMG findings (indicating no evidence of NMJ dysfunction) significantly reduced suspicion for NMJ disorders. Furthermore, the overall clinical progression, i.e., ascending weakness with respiratory failure and partial clinical response to IVIG, strongly favoured a peripheral nerve aetiology, such as AMAN or post-infectious autoimmune neuropathy, over an NMJ or muscle pathology. This aligns with the known variability and diagnostic uncertainty in atypical immune-mediated neuropathies [2,3].

Despite the negative findings for demyelination, empirical treatment with IVIG led to clinical improvement, specifically the normalization of F-wave persistence in NCS. F-waves are valuable in diagnosing GBS, as they reflect the excitability of motor neurons and can indicate conduction block or slowed conduction, serving as a sensitive electrophysiological marker in atypical or early-stage GBS cases [3,4]. The improvement in F-wave persistence observed following IVIG administration suggests a possible reduction in inflammation or immune-mediated nerve dysfunction, even in the absence of classical GBS features. Similar effects of IVIG on motor nerve excitability and F-wave normalization have been noted in other immune-mediated neuropathies, such as multifocal motor neuropathy, although specific evidence in GBS remains limited [5].

Interestingly, the patient reported a sudden flare-up of vitiligo before the onset of her neurological symptoms. Vitiligo is an autoimmune disorder characterized by immune-mediated destruction of melanocytes, resulting in depigmented skin patches [6]. An exacerbation of vitiligo may indicate increased systemic autoimmune activity, potentially correlating with broader immune-mediated mechanisms affecting the nervous system [6,7]. Several studies have demonstrated associations between vitiligo and other systemic autoimmune conditions, including neurological disorders such as multiple sclerosis and systemic lupus erythematosus, highlighting how cutaneous manifestations can precede or coincide with neurological symptoms [7,8]. This suggests that the neurological presentation in this patient might share an underlying autoimmune mechanism with her dermatological symptoms.

The development of respiratory failure requiring intubation and eventual tracheostomy highlights the severity and objective nature of her neurological deterioration, making a purely psychogenic diagnosis unlikely. Although psychogenic causes were initially considered, particularly during the early phase when neurological signs were subtle and objective evidence was less definitive, the rapid progression to respiratory compromise, facial muscle weakness, and measurable deficits on spirometry objectively indicated an organic pathology rather than a functional disorder [9,10]. Additionally, the partial but significant therapeutic response to IVIG further supported an immune-mediated process. Autoimmune aetiologies remained highly probable, particularly an atypical autoimmune neuropathy or a post-infectious immune response, consistent with the spectrum of disorders that present similarly to GBS but lack definitive electrophysiological evidence [1,2]. Reports in the literature highlight similar clinical presentations in atypical immune-mediated neuropathies, where the absence of diagnostic biomarkers complicates definitive classification, and empirical immunotherapy often guides diagnosis and management [1-3].

The absence of demyelinating features on MRI and the lack of typical findings on repeated EMG/NCS studies argue against classical GBS or its variants [11]. Alternative conditions, including CIDP, autoimmune encephalitis, and paraneoplastic syndromes, can present similarly and respond to immunotherapy, highlighting the diagnostic complexity [11,12]. A comprehensive autoimmune and neuromuscular panel, including AChR, MuSK, leucine-rich glioma-inactivated 1 (LGI1), N-methyl-D-aspartate (NMDA) receptor, voltage-gated calcium channels (VGCC), and ganglioside (GM1) antibodies, and paraprotein screening (serum electrophoresis), was negative. Additionally, imaging, including MRI of the brain and spinal cord and CT of the chest, was unremarkable except for features attributable to aspiration pneumonia. Given these negative results and clinical improvement with IVIG, further extensive paraneoplastic screening or biopsies were not pursued initially but may be reconsidered if symptoms recur. This underscores the importance of maintaining a broad differential diagnosis in atypical immune neuropathies that may not align neatly with classical patterns but still demonstrate therapeutic response to immunomodulatory treatments [11,12]. Moving forward, the patient will undergo ongoing neurological assessment, with plans for repeat evaluation of autoimmune and paraneoplastic markers, neuroimmunology panels, or targeted imaging if symptoms re-emerge.

Given the patient’s South Indian ethnicity, genetic and environmental factors that influence autoimmune susceptibility should also be considered. Certain human leukocyte antigen (HLA) alleles prevalent in South Asian populations have been associated with increased susceptibility to autoimmune diseases such as rheumatoid arthritis and systemic lupus erythematosus [13]. Additionally, vitamin D deficiency, which is common among South Indian women due to limited sun exposure, has been implicated in immune dysregulation, increasing the risk of autoimmune disorders, including multiple sclerosis and rheumatoid arthritis [14,15]. Furthermore, exposure to tropical infections may serve as a potential trigger for autoimmune responses in genetically predisposed individuals [16]. These factors collectively emphasize the complex interplay of genetic predisposition and environmental influences in autoimmune disease pathogenesis.

Limitations in this case include the possibility that early intervention with IVIG may have masked characteristic electrophysiological abnormalities typically seen on NCS and EMG, as well as the absence of specific biomarkers confirming an autoimmune aetiology. Despite these limitations, the observed clinical improvement following IVIG treatment provides supportive evidence for an immune-mediated mechanism, though definitive diagnostic conclusions require additional studies or longer follow-ups for further clarification.

## Conclusions

This case underscores the challenges in diagnosing and managing acute neurological presentations when classical diagnostic criteria are not fully met. Although GBS was initially suspected, the absence of typical EMG and NCS findings, along with normal MRI, argued against this diagnosis. However, the patient's positive clinical response to IVIG supports an immune-mediated process, aligning with atypical autoimmune neuropathies. Additionally, the flare-up of vitiligo preceding neurological symptom onset suggests heightened systemic autoimmune activity, highlighting the importance of recognizing cutaneous autoimmune manifestations as potential indicators of neurological involvement.

This case emphasizes the value of a comprehensive, multidisciplinary approach in diagnostically ambiguous presentations, particularly when ethnic and regional factors might influence disease susceptibility. In South Indian populations, genetic predispositions (such as specific HLA alleles), prevalent vitamin D deficiency, and tropical infectious exposures may collectively influence autoimmune disease risk, warranting further investigation. Given the diagnostic uncertainty, continued close neurological monitoring and preservation of CSF and serum samples are essential to facilitate future testing if symptoms recur or evolve. Ultimately, this case supports empirical immunotherapy in suspected immune-mediated aetiologies, even when definitive diagnostic markers are absent. Further research exploring associations between systemic autoimmune activity and neurological disorders could significantly enhance diagnostic accuracy and therapeutic outcomes.
